# Frequency of non-single canals in mandibular premolars and correlations with other anatomical variants: an in vivo cone beam computed tomography study

**DOI:** 10.1186/s12903-019-0972-5

**Published:** 2019-12-04

**Authors:** Young-Eun Jang, Yemi Kim, BomSahn Kim, Sin-Young Kim, Hyung-Jong Kim

**Affiliations:** 1 Department of Conservative Dentistry, College of Medicine, Ewha Womans University, 1071, Anyangcheon-ro, Yangcheon-gu, Seoul, 07985 South Korea; 2 Department of Nuclear Medicine, College of Medicine, Ewha Womans University, Seoul, South Korea; 3 Department of Conservative Dentistry, Seoul St. Mary’s Dental Hospital, College of Medicine, The Catholic University of Korea, Seoul, South Korea

**Keywords:** Root canal configuration, Mandibular premolars, Cone beam computed tomography, C-shaped root canal system, Distolingual roots, Korean population

## Abstract

**Background:**

A knowledge regarding anatomical variants is important to achieve success in endodontic treatment. Root canal treatment of mandibular first premolars (PM1 s) is challenging due to the existence of numerous variations in canal configurations, including a C-shaped variant. We aim to determine the frequency and morphologic characteristics of non-single canals of mandibular first (PM1 s) and second (PM2 s) premolars in a Korean population using cone beam computed tomography (CBCT) and to evaluate correlations between non-single canals of PM1 s and other anatomical variants, such as distolingual roots (DLRs) in mandibular first molars (M1 s) and C-shaped canals in mandibular second molars (M2 s).

**Methods:**

A total of 971 PM1 s and 997 PM2 s from 500 patients were examined in vivo by CBCT. Root canal configurations and C-shaped canals were determined in accordance with the Vertucci classification and Fan classification, respectively. The correlation between non-single canals in PM1 s and DLRs in M1 s was evaluated using logistic regression analysis.

**Results:**

PM2 s typically had one root (99.89%) with one canal (98.4%). Among PM1 s with non-single canals (21.2%), Vertucci type V (10.9%) and C-shaped (3.7%) canals were prevalent. Among C-shaped PM1 canals, the majority were Vertucci type V (77.8%); a C-shaped configuration (C2) was predominant mostly at the middle and/or apical third of the root. After adjusting for other variables (i.e., sex, age, and side), C-shaped canals in PM1 s was significantly correlated with the presence of DLRs in M1 s (odds ratio = 2.616; 95% confidence interval, 1.257–5.443; *p* = 0.010).

**Conclusions:**

The presence of C-shaped PM1 canals was positively related to the presence of DLRs in M1 s. Although C-shaped canals in PM1 s are difficult to distinguish, this finding could aid clinicians in predicting C-shaped canal configurations in PM1 s of patients who exhibit DLRs in M1 s.

## Background

A lack of knowledge regarding anatomical variants may result in untreated canal space, potentially leading to endodontic treatment failure [1]. Mandibular premolars often exhibit complex anatomy that cannot be clearly detected in two-dimensional periapical radiographs [2, 3]. Previous studies have shown high frequencies of non-single canal systems in mandibular premolars (12.9–34.8% and 2–9.9% in mandibular first and second premolars, respectively) [4–9]. These variations are caused by differences in methodology, ethnicity, and characteristics of participants, such as age and sex [4, 5].

In vivo cone beam computed tomography (CBCT) has comparable accuracy to that of micro-computed tomography (micro-CT) for detecting root canal morphology [6]. Previous studies have suggested that CBCT analysis could be useful for determining root canal anatomy [7–12]. Notably, its noninvasive application allows collection of a greater number of samples than in previous studies that were limited to the use of extracted teeth; these samples are thus sufficiently large to be representative of the general population. In addition, differences in sex, side, and relationships with other variations in root canal morphology can be easily compared in CBCT scans.

Endodontic treatment of mandibular first premolars (PM1 s) is challenging due to the existence of numerous variations in canal configurations, including a C-shaped variant; moreover, the relatively small diameter of PM1 s limits direct access to additional canals [13, 14]. To the best of our knowledge, little information is available regarding the frequency of C-shaped canals in PM1 s (as shown in CBCT images) and their correlations with other anatomical variants, such as distolingual roots (DLRs) in mandibular first molars (M1 s) and C-shaped canals in mandibular second molars (M2 s). Therefore, this study aimed to retrospectively investigate the roots and root canal configurations in PM1 s and PM2 s in a Korean population using a large number of CBCT images and to assess correlations between non-single canals in PM1 s and other anatomical variations, including DLRs in M1 s and C-shaped canals in M2 s.

## Methods

### Study design

The protocol of this retrospective, cross-sectional study was approved by the Ethics Committee of Ewha Womans University Hospital, Seoul, Korea (no. EUMC 2018–01-064). Images of mandibular premolars were obtained from patients who had undergone CBCT scanning at the hospital between January 2011 and November 2012. CBCT images were acquired using a Dinnova system (Willmed, Gwangmyeong, Korea) with the following parameters: 80 kVp, 9.0 mA, 10 × 10-cm field of view, 0.167-mm^3^ voxel size, and the slice thickness was 1.0 mm. Cross-sectional images in the axial, coronal, and sagittal planes were reconstructed using OnDemand3D software (Cybermed, Seoul, Korea). CBCT scans were generally acquired for implant surgery or surgical removal of impacted molars. Therefore, no subjects in this study were exposed to unnecessary radiation to obtain information regarding root canal anatomy; moreover, the “as low as reasonably achievable” principle was followed with respect to radiation dose. Before examining the images, data were anonymized by numbering the subjects from 1 to 500 to prevent any possible bias.

### Study size and subjects

The required study size was determined using PASS software (ver. 13.0; NCSS, Kaysville, UT, USA). The Korean population constitutes approximately 50 million people; with a margin of error of 5% and confidence levels of 98 and 97%, the required sample sizes were 542 and 471, respectively. Thus, we retrospectively included 500 subjects from among 1393 patients who met the following inclusion criteria:

1. Age between 13 and 69 years;
2. Imaging data available, including scans of fully erupted mandibular premolars;
3. Presence of mandibular premolars with fully matured apices and without apical periodontitis;
4. Presence of mandibular premolars without root canal fillings, posts, crown restorations, or any metallic restorations;
5. Patients undergoing orthodontic treatment; and
6. Patients with calcified canals

### Image assessment

Serial axial-, coronal-, and sagittal-plane CBCT images were closely examined at 1.0-mm intervals from the canal orifice to the apex. The numbers of roots and root canals were recorded, as were the configurations of the root canals and the frequencies of unilateral and bilateral root canal configurations.

The numbers of roots in PM1 s were determined by examining axial-plane images. Single-rooted teeth had conical-shaped roots; these included teeth with two canals with a fused root. Double-rooted teeth exhibited bifurcation at a certain root level; these included teeth with two canals in a single fused root and a third canal in a separate root. Triple-rooted teeth exhibited three independent roots. The presence of DLRs in M1 s and C-shaped M2 s was also examined in axial-plane images.

Root canal configurations were determined in accordance with the Vertucci classification [15]. Thus, root canals other than Vertucci type I were regarded as “non-single canals”; root canals with more than one canal, except C-shaped canals, were regarded as “complicated canals.”

C-shaped canal configurations were determined in accordance with the Fan classification [16], as follows: C1 (continuous C-shaped canal: an uninterrupted “C” without separation or division); C2 (semicolon-shaped canal: caused by discontinuation of the “C” outline); C3 (separated canals: two or three separate canals); C4 (a single canal subdivided into round (C4a), oval (C4b), or flat canals (C4c)); C5 (≥3 separate canals); or C6 (no visible canal lumen). The distribution of radicular grooves was noted. The presence of a C1 or C2 configuration at any position of the root canal was taken to indicate a C-shaped root canal system.

All images were independently assessed by two endodontists who were experienced in CBCT imaging. Both experts viewed the images on a 27-in. monitor (SE2717H; Dell, Round Rock, TX, USA) with a screen resolution of 1920 × 1080 and 32-bit color depth and used same CBCT software. They were examined by carefully rolling the toolbar from the canal orifice to the apex and navigated freely through CBCT volumes. Kappa values for intra- and interobserver reliability were calculated by evaluating 60 randomly selected images twice, with an interval of 1 week between evaluations; intra- and interobserver values were 0.85 and 0.79, respectively, which were considered adequate. After calibration, all study subjects were examined independently. The examiners magnified the images as necessary for proper assessment of both root and root canal morphology. In instances of disagreement, images were re-evaluated and discussed until a consensus was reached.

### Statistical analysis

Statistical analyses were performed using SPSS software (ver. 21; SPSS, Inc., Chicago, IL, USA). The chi-squared test was performed for analyses of differences based on sex, tooth location (left or right side), and bilateral PM1 canal configurations. The chi-squared test was also used to compare the frequencies of DLRs in M1 s and C-shaped canals in M2 s according to PM1 root canal configuration and to compare unilateral and bilateral M1 DLRs and M2 C-shaped canal configurations.

To evaluate correlations between root canal configurations and other anatomical variants, multivariate logistic regression and multinomial logistic regression analyses were used, with adjustments for sex, age, and side. Odds Ratio (OR) along with 95% Confidence Interval (CI) was used to assess statistical significance of associations. Multivariate logistic regression analysis was used to assess correlations of root canal configurations (single and non-single canals) in PM1 s with other anatomical variants. Multinomial logistic regression analysis was used to evaluate correlations of root canal configurations (single, complicated, and C-shaped canals) in PM1 s with other anatomical variants.

## Results

### Frequency of premolars with non-single canals

**Fig. 1:**
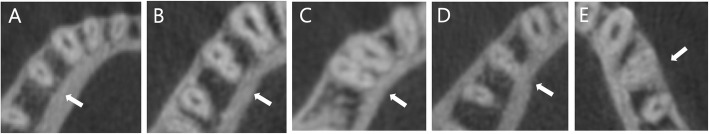
Axial view of root canal configurations of mandibular first premolars; arrows indicate the examined teeth. **a** One conical root. **b**, **c** One root with two canals. **d** Two roots with two canals. **e** Two roots with three canals

From among the 500 patients (252 women and 248 men; mean age, 28.61 ± 10.02 years), a total of 1968 premolars were examined (971 PM1 s and 997 PM2 s). The majority of PM1 s had one root (97.9%) and one canal (78.78%) (Table 1). Among PM1 s with ≥2 root canals (21.2%), Vertucci type V (10.9%) and C-shaped (3.7%) canals were more frequent than other types. C-shaped anatomy was apparent primarily in Vertucci type V canals (77.8%) (Table 2). Axial views of PM1 roots and root canal configurations are shown in Fig. 1. Most PM2 s had one root (100%) and one canal (98.4%). The frequencies of > 1 canal (1.6%) and of C-shaped root canals (0%) were lower in PM2 s than in PM1 s.

**Table 1:** Frequencies of the root and root canal configurations of mandibular premolars

	Root morphology	Root position	Number of canals	Mandibular first premolar	Mandibular second premolar
1 root (*n* = 1947)	Conical		1 canal	765 (78.78)	981 (98.4)
			2 canals	100 (10.3)	16 (1.6)
			3 canals	0 (0)	0 (0)
	Fused	B, L	2 canals	50 (5.15)	0 (0)
	C-shaped			36 (3.7)	0 (0)
2 roots (*n* = 21)	2S	B, L	2 canals	14 (1.44)	0 (0)
	1F 1S	MB, DB & L	3 canals	6 (0.62)	0 (0)
Total				971 (100)	997 (100)

S, separate; F, fused

**Table 2:** Root canal morphologies of mandibular premolars

		Vertucci classification, n (%)											
		I	II	III	IV	V	VI	VII	VIII	Additional		Others	C-shape
		1–1	2–1	1–2-1	2	1–2	2–1-2	1–2–1-2	3	2–3	3–2		
Mandibular first premolar (*n* = 971)		765 (78.78)	22 (2.27)	31 (3.19)	4 (0.41)	106 (10.92)	0 (0)	1 (0.1)	0 (0)	1 (0.1)	5 (0.51)		36 (3.71)
	C-shape (*n* = 36)	–	–	5 (13.9)	–	28 (77.8)	–	–	–			3 (8.3)	
Mandibular second premolar (*n* = 997)		981 (98.4)	14 (1.4)	2 (0.2)	–	–	–	–	–				–

### Canal configuration by sex and bilateral distribution of root canal configuration in PM1 s

Compared with women, men more frequently showed complicated canal configurations and C-shaped canals in PM1 s (*p* <  0.001; Table 3). No left- or right-sided predominance was detected among Vertucci root canal configurations or C-shaped canals (*p* = 0.95). In PM1 s, the respective frequencies of bilateral root canal configuration for a single canal, complicated canals, and C-shaped canals were 93.2% (700 of 751), 69% (116 of 168), and 62.9% (22 of 35). The overall bilateral frequency rate was 87.8% (838 of 954 canals, *p* <  0.001). Of the complicated canals on both sides (69%), 60.7% of root canals had the same Vertucci type, and 8.3% had different Vertucci types. With respect to unilateral C-shaped canals, more than half of the contralateral canals were complicated canals (Table 3).

**Table 3:** Canal configuration frequencies of mandibular first premolars by sex and root canal configurations by unilateral or bilateral status

Mandibular first premolar	Sex			Bilateral		Unilateral						
						Left	Right		Contralateral			Subtotal
	Female	Male	*P* value	n	*P* value	n	n	*P* value	Single	Complicated	C-shaped	n
Single (*n* = 751)	419 (84.8)	332 (72.2)	<.001*	700 (93.2)	<.001*	29 (50)	22 (37.9)	.095	–	45 (88.2)	6 (11.8)	51 (6.8)
Complicated (*n* = 168)	65 (13.2)	103 (22.4)		116 (69)		26 (44.8)	26 (44.8)		45 (86.5)	–	7 (13.5)	52 (31)
C-shaped (*n* = 35)	10 (2)	25 (5.4)		22 (62.9)		3 (5.2)	10 (17.2)		6 (46.2)	7 (53.8)	–	13 (37.1)
Total (*n* = 954)	494 (51.8)	460 (48.2)		838 (87.8)		58 (6.1)	58 (6.1)		51 (6.8)	52 (31)	13 (37.1)	116 (12.2)

### C-shaped canals in PM1 s

**Fig. 2:**
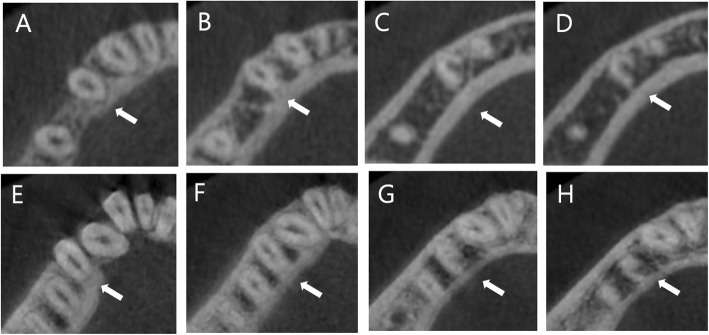
Axial view of C-shaped canal configurations of mandibular first premolars; arrows indicate the examined teeth. **a**, **e** Level of orifice. **b**, **f** Level of coronal third. **c**, **g** Level of middle third. **d**, **h** Level of apical third

All C-shaped PM1 canals showed a radicular groove or concavity of the external root surface; most grooves (94.4%) were on the mesiolingual surface of the root. Root canal configuration varied with respect to vertical position in the root. In the coronal third of the root, most canals were single canals (C4) (Table 4); a C2 configuration was present mostly in the middle and/or apical thirds. An axial view of a C-shaped root PM1 canal is shown in Fig. 2.

**Table 4:** Distributions of C-shaped canal configurations in C-shaped mandibular first premolars according to root level

	Cross-sections of C-shaped canals, N (%)		
	Coronal	Middle	Apical
C1	–	–	–
C2	–	27 (75)	15 (41.7)
C3	–	7 (19.4)	16 (44.4)
C4a	7 (19.4)	2 (5.6)	2 (5.6)
C4b	16 (44.4)		3 (8.3)
C4c	13 (36.1)		
Total	36 (100)	36 (100)	36 (100)

### Correlations of non-single PM1 canals with other anatomical variants

**Fig. 3:**
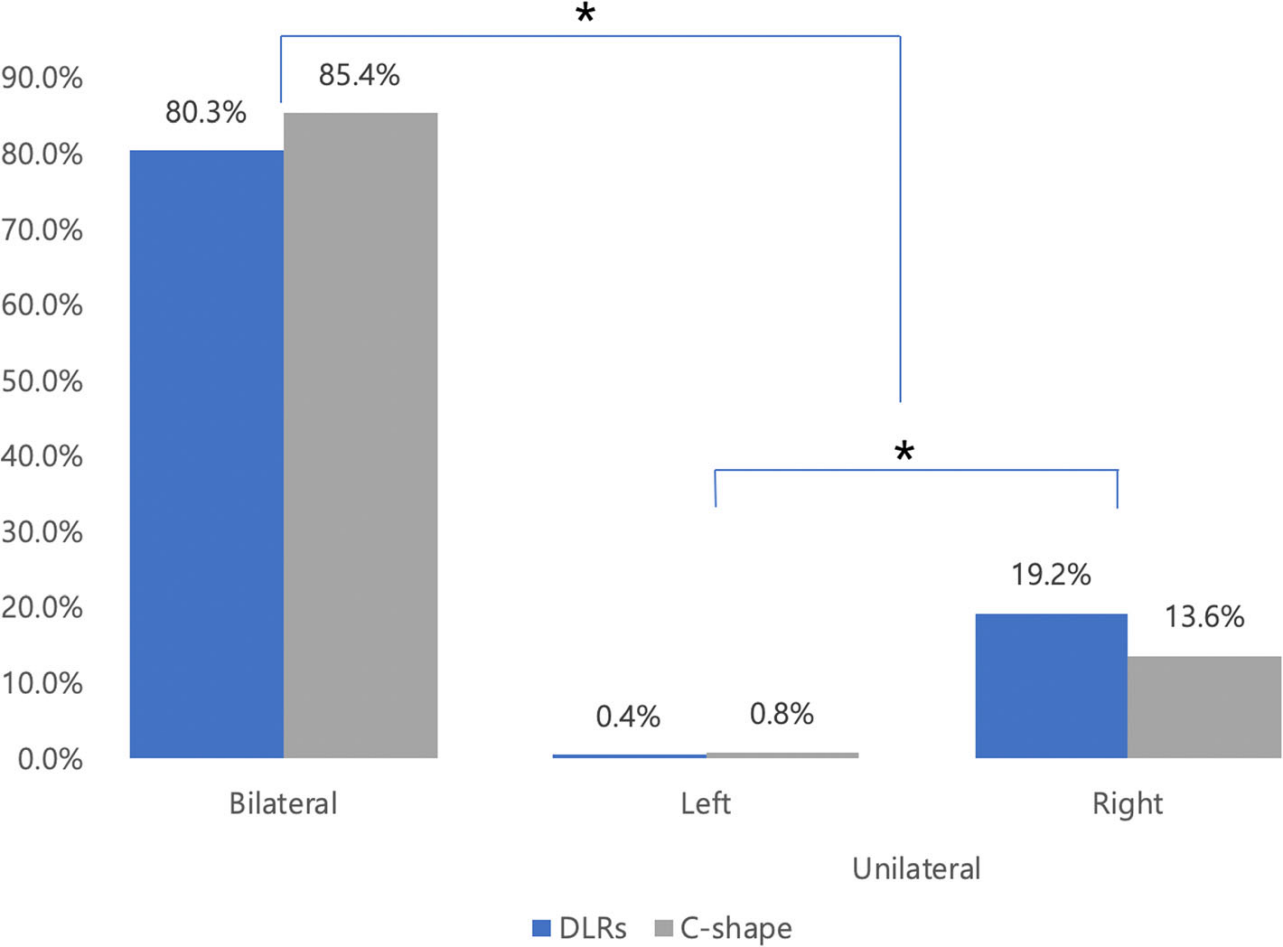
Frequencies of distolingual roots in mandibular first molars and C-shaped canal configurations in mandibular second molars according to unilateral or bilateral status

The respective frequencies of DLRs in M1 s and C-shaped canals in M2 s were 25.4% (229 of 902) and 43.8% (395 of 902). Significant bilateral symmetry was noted in M1 s with DLRs (80.3%, 184 of 229; *p* <  0.001) and C-shaped M2 s (85.6%, 338 of 396; *p* <  0.001). The incidence of DLRs in M1 s was significantly greater on the right side (44 of 45; *p* < 0.001, Fig. 3).

**Fig. 4:**
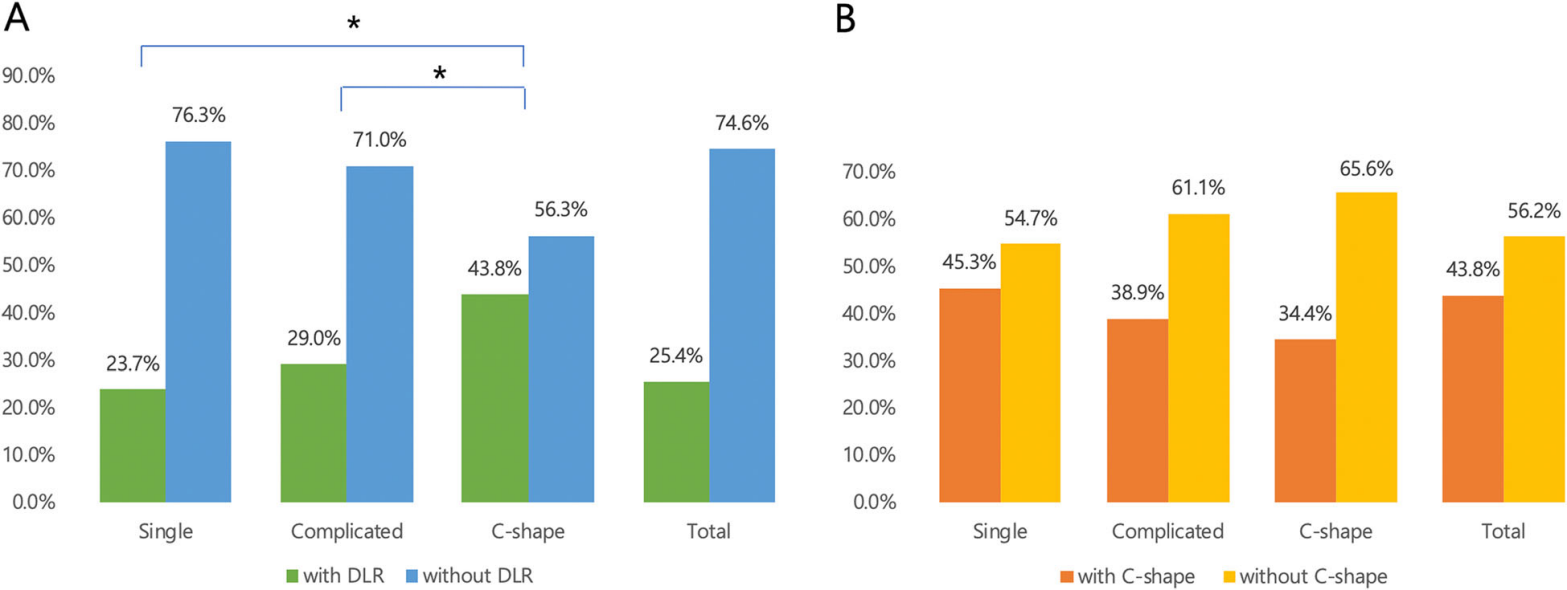
Associations of root canal configurations of mandibular first premolars with **a** distolingual roots in mandibular first molars and **b** C-shaped canals in mandibular second molars

Chi-squared analysis revealed that the frequency of M1 DLRs varied significantly according to root canal configuration (*p* < 0.05), whereas the frequency of C-shaped M2 s did not (*p* = 0.181, Fig. 4). Thus, logistic regression analysis was used to assess the correlation between M1 DLRs and PM1 root canal configuration.

Multivariate logistic regression analysis showed that, after adjusting for sex, age, and side, the presence of non-single PM1 canals was significantly correlated with the presence of DLRs in M1 (odds ratio [OR] = 1.539; 95% confidence interval [CI], 1.077–2.200; *p* = 0.018, Table 5). Multinomial logistic regression analysis showed that, after adjusting for sex, age, and side, the presence of C-shaped canals in PM1 s was significantly correlated with the presence of DLRs in M1 s (OR = 2.616; 95% CI, 1.257–5.443; *p* = 0.010, Table 6); conversely, the presence of complicated canals in PM1 s was not associated with the presence of DLRs in M1 s (*p* = 0.107, Table 6). The frequency of C-shaped canals in PM1 s was positively related to the bilateral presence of DLRs in M1 s (*p* < 0.05, Table 7).

**Table 5:** Multivariate logistic regression analyses of the frequencies of non-single canal configurations in mandibular first premolars

Explanatory variables (test category/reference category)	Multivariate logistic regression analyses	
	Non-single canal	
	Odds ratio (95% CI)	*P* value
Sex (male/female)	2.281 (1.637–3.179)	< 0.001*
Age (per year)	0.988 (0.971–1.005)	0.156
Side (left/right)	0.987 (0.713–1.367)	0.939
DLRs in M1 s (yes/no)	1.539 (1.077–2.200)	0.018*

Complicated canals and C-shaped canals were categorized as non-single canal

*The significance level is *P* = .05

**Table 6:** Multinomial logistic regression analyses of the frequencies of complicated canals and C-shaped canals in mandibular first premolars

Explanatory variables (test category/reference category)	Multinomial logistic regression analyses			
	Complicated canal		C-shaped canal	
	Odds ratio (95% CI)	*P* value	Odds ratio (95% CI)	*P* value
Sex (male/female)	2.166 (1.520–3.087)	< 0.001*	3.039 (1.404–6.580)	0.005*
Age (per year)	0.991 (0.973–1.009)	0.324	0.969 (0.929–1.010)	0.136
Side (left/right)	1.042 (0.736–1.475)	0.817	0.740 (0.356–1.537)	0.419
DLRs in M1 s (yes/no)	1.376 (0.933–2.019)	0.107	2.616 (1.257–5.443)	0.010*

*The significance level is *P* = .05

**Table 7:** Distribution of C-shaped canal configuration in mandibular first premolars according to the unilateral or bilateral presence of distolingual roots in mandibular first molars

	Mandibular first molar				
	With DLR				
	Bi-DLR	Uni-DLR	Non-DLR	Total	*P* value
C-shape canals in PM1 s					
Yes	12 (6.5)	2 (4.4)	18 (2.7)	32 (3.5)	.042*
No	172 (93.5)	43 (95.6)	655 (97.3)	870 (92.5)	
Total	184 (100)	45 (100)	673 (100)	902 (100)	

*The significance level is *P* = .05

## Discussion

This in vivo retrospective study used CBCT scanning to investigate the root and root canal morphology of premolars, as well as correlations between non-single canals of premolars and other anatomical variants (i.e., DLRs in M1 s and C-shaped canals in M2 s). The frequency of PM1 s with a single canal (760 of 971, 78.27%) was comparable to those reported in two systematic reviews (75.8 and 73.55%) [17, 18], as well those reported in East Asian populations (e.g., Chinese and Taiwanese [65.2–87.1%]) [7, 8, 19–21]. Regarding the root canal morphology of PM2 s, a recent review reported a markedly lower incidence of a second canal (2%) in East Asian populations compared with other populations [18]. This finding agreed with our results, which showed that only 1.6% of PM2 s contained two canals. In the present study, men had significantly more root canals and C-shaped canals in PM1 s than did women (Table 3); this is also consistent with previous results [9, 11, 19, 22, 23]. The findings regarding anatomical conditions and sex showed were conflicting [12, 24]. Bilateral root canal configurations were noted in a significantly higher proportion of PM1 s (85.9%, *p* < 0.05; Table 3), in agreement with the findings of previous studies [12, 19]. In the present study, when complicated or C-shaped canals were observed in PM1 s, the canals were bilateral in 68% of subjects. Thus, when non-single canals are present in PM1 s, clinicians should consider the possibility of complicated canals in contralateral premolars.

In PM1 s with two canals, our results indicated that the Vertucci type V configuration was more prevalent than the other types. This is consistent with the findings of previous in vivo studies that analyzed CBCT images of Chinese [7, 8, 21], German [9], and Turkish populations [11, 25], as well as with the findings of an in vitro study that analyzed the micro-CT data of a Chinese cohort [20]. However, our results are not consistent with the findings of other in vitro studies [26, 27], which showed that other Vertucci types (i.e., II or IV) were more prevalent. These variations in root canal morphology may reflect differences in ethnicity, age, sex, and/or research methodology [5, 17]. Although no consensus has been reached regarding ethnic differences in the most common internal canal configuration of complicated canals in PM1 s, a recent study indicated that among complicated canals, Vertucci type V was most frequent in both Asian (12.6%) and White ethnic groups (12.2%) [4]. Information regarding the most common internal root canal morphology of PM1 s could help clinicians to anticipate bifurcation at the middle third of PM1 roots with a single coronal canal.

The frequency of C-shaped root canal systems was 3.6% in our study, which was consistent with that of previous studies in which CBCT analysis was used [7, 8]. PM1 s typically cause the greatest difficulty for clinicians; moreover, these show the highest rates of failure after root canal treatment [28]. This might be due to the complexity of the root canal morphology and the appearance of the orifice in C-shaped mandibular premolars. In the present study, the canal configuration of mandibular C-shaped premolars was typically oval at the coronal third of the root (C4 configuration), whereas the C configuration was observed mainly at the middle third (Table 5). Our findings are in agreement with those of previous studies [13, 29, 30], which [17, 30, 31] reported that C-shaped canals were primarily located in the apical half of the root. However, previous investigations of C-shaped canals in M2 s showed that the majority of the canal orifices had a continuous C-shape or an incomplete C configuration, whereas 0–9% were round or oval in shape [16, 31]. The coronal oval canal is a distinguishing characteristic of C-shaped canals in mandibular premolars. Clinicians should be aware of this feature and should not define canal configuration based on coronal canal morphology. For straight-line access, the use of an operating microscope is recommended to detect bifurcation and establish whether the orifice extends in the buccolingual direction [13, 14, 17, 18].

Our results regarding the frequency of DLRs in M1 s (25.3%) are consistent with those of previous studies in Asian populations (22–25.9%) [4, 12, 32]. A recent study showed a positive correlation between in M1 DLRs and complicated PM1 canal configurations [12]. In that study, C-shaped canals were categorized as complicated canals, a major difference relative to our study. In the present study, we confirmed a positive correlation between non-single PM1 canals and DLRs in M1 s. We then subdivided non-single canals into complicated and C-shaped canal configurations. We found that the presence of C-shaped canals was significantly correlated with the presence M1 DLRs (*p* = 0.010; Table 6), whereas complicated canals did not show a significant relationship with in M1 DLRs (*p* = 0.107). After adjusting for sex, age, and side, we found that the frequency of C-shaped canals was 2.616-fold greater than that of single canals in subjects with DLRs. In addition, the presence of C-shaped PM1 canal configurations was significantly more frequent when bilateral DLRs were present (*p* < 0.05; Table 7). As noted above, C-shaped PM1 canal configurations may be difficult to recognize due to the coronal appearance, with bifurcation in the middle and/or apical third of the root. This finding of a correlation between C-shaped PM1 canals and M1 s DLRs could provide an advantage to clinicians, as they might anticipate the possibility of C-shaped canals in PM1 s when DLRs are observed.

There was no correlation between non-single PM1 canals and C-shaped M2 canals (*p* > 0.05). Notably, the frequency of C-shaped canals in M2 s is very high (43.8%) in the Korean population. Clinicians should note that C-shaped canal configurations are commonly found in M2 s in the Korean population regardless of the presence of C-shaped canals in PM1 s. Although CBCT imaging is somewhat less accurate to detect sophisticated root canal anatomy (e.g., multiple apical foramen or accessory canal) compared to micro-CT, CBCT is recommended for future in vivo studies in different regions/races to investigate the true frequencies and morphologic characteristics of various root canal configurations and their correlations with other anatomical variants.

## Conclusions

This retrospective in vivo study showed that more than one-fifth (21.2%) of PM1 s in a Korean population had ≥2 root canals. The most common canal configurations were Vertucci type V (10.9%), followed by C-shaped canals (3.7%); the frequency of complicated canals in PM2 s was lower (1.6%). We found that the presence of C-shaped PM1 canals was significantly correlated with the presence of DLRs in M1 s. This finding could help clinicians to predict the presence of C-shaped canal configurations in the PM1 s of patients who exhibit DLRs in M1 s.

## Data Availability

The datasets used and/or analyzed during the current study are available in the Zenodo repository [https://zenodo.org/record/3332055#.XSck3S3SiqA].

## Abbreviations

CBCT: Cone-beam computed tomography
DLRs: Distolingual roots
M1 s: Mandibular first molars
M2 s: Mandibular second molars
PM1 s: Mandibular first premolars
PM2 s: Mandibular second premolars
